# Temperature Switch PCR (TSP): Robust assay design for reliable amplification and genotyping of SNPs

**DOI:** 10.1186/1471-2164-10-580

**Published:** 2009-12-03

**Authors:** Tania Tabone, Diane E Mather, Matthew J Hayden

**Affiliations:** 1 Molecular Plant Breeding Co-operative Research Centre and School of Agriculture, Food and Wine, The University of Adelaide, Glen Osmond, SA 5064, Australia; 2 Ludwig Institute for Cancer Research, Royal Melbourne Hospital, Parkville, VIC 3050, Australia; 3 Department of Primary Industries Victoria, Victorian AgriBiosciences Centre, La Trobe R&D Park, Bundoora, VIC 3083, Australia

## Abstract

**Background:**

Many research and diagnostic applications rely upon the assay of individual single nucleotide polymorphisms (SNPs). Thus, methods to improve the speed and efficiency for single-marker SNP genotyping are highly desirable. Here, we describe the method of temperature-switch PCR (TSP), a biphasic four-primer PCR system with a universal primer design that permits amplification of the target locus in the first phase of thermal cycling before switching to the detection of the alleles. TSP can simplify assay design for a range of commonly used single-marker SNP genotyping methods, and reduce the requirement for individual assay optimization and operator expertise in the deployment of SNP assays.

**Results:**

We demonstrate the utility of TSP for the rapid construction of robust and convenient endpoint SNP genotyping assays based on allele-specific PCR and high resolution melt analysis by generating a total of 11,232 data points. The TSP assays were performed under standardised reaction conditions, requiring minimal optimization of individual assays. High genotyping accuracy was verified by 100% concordance of TSP genotypes in a blinded study with an independent genotyping method.

**Conclusion:**

Theoretically, TSP can be directly incorporated into the design of assays for most current single-marker SNP genotyping methods. TSP provides several technological advances for single-marker SNP genotyping including simplified assay design and development, increased assay specificity and genotyping accuracy, and opportunities for assay automation. By reducing the requirement for operator expertise, TSP provides opportunities to deploy a wider range of single-marker SNP genotyping methods in the laboratory. TSP has broad applications and can be deployed in any animal and plant species.

## Background

The availability of comprehensive collections of genomic and EST information for human and many species of animals and plants, and the development of technologies for the rapid resequencing of specific genomic regions have paved the way for the routine application of single nucleotide polymorphisms (SNPs) as DNA markers. The methods currently available for SNP genotyping provide a continuum for assay scalability ranging from single-marker diagnostic assays to genome-wide scans, in which thousands of SNPs are assayed in parallel [1,2]. While genome-wide scans are useful for association studies and the initial discovery of target loci, it is the single-marker assays that are most useful in the latter stages of research and for diagnostic applications.

Numerous single-marker methods have been developed to genotype SNPs. Many of these methods are based on oligonucleotide ligation and allele-specific primer extension chemistry, as they do not require dedicated equipment. These methods include tetra-primer PCR [3], PCR amplification of specific alleles (PASA) [4], amplification refractory mutation system (ARMS) [5], oligonucleotide ligation assay (OLA) [6] and padlock probes [7]. Other methods are based on detecting changes to the physical properties of DNA such as high resolution melt [8], single-strand conformation polymorphism [9] and denaturing high performance liquid chromatography [10], or rely on enzymatic modification such as PCR-RFLP [11], 5' nuclease (TaqMan^®^) [12] and Invader^® ^[13] assays. Regardless of the assay principles, these methods share the common feature that a pair of oligonucleotide probes is designed to cover and flank the SNP. These probes may be used to amplify the target region and manipulate the location of the polymorphism to an optimal position within the PCR fragment that maximises detection sensitivity. Alternatively, at least one of the probes may be designed either adjacent to, or positioned over, the polymorphism for direct interrogation of the SNP. Although most types of sequence polymorphism are amenable to assay design for at least one of these genotyping methods, it is not always possible to use a single genotyping method to assay a panel of SNPs. Besides the nature of the SNP iteslf, other factors that impede assay design for a particular genotyping method include the sequence composition adjacent to the SNP, the presence of repetitive DNA, or sequence homology with related genes. Hence, laboratories must typically deploy a suite of genotyping methods.

Successful integration of any SNP genotyping method into the laboratory depends on the ability to rapidly develop and implement new assays. This is especially important in genetic research and diagnostic contexts in which genotyping demands vary over time. One of the major limitations to the integration of many of these methods is the requirement to individually optimize each SNP assay, the success of which is often dependent on the expertise of the operator. Slow assay development can impact on genotyping throughput, while lack of expertise for particular methods can preclude the use of the optimal method for each SNP.

Here, we describe the method of temperature-switch PCR (TSP), which was developed to address difficulties that are commonly associated with deploying a variety of single-marker SNP genotyping methods in the laboratory. TSP can simplify assay design for a range of commonly used single-marker SNP genotyping methods and reduce the need for individual assay optimization. By reducing the requirement for operator expertise, TSP provides opportunities to deploy a wider range of single-marker SNP genotyping methods in the laboratory. The present study demonstrates the utility of TSP for the rapid development of robust endpoint SNP genotyping assays based on allele-specific PCR and high resolution melt analysis in cultivated barley (*Hordeum vulgare *L.), an agriculturally important crop with a 5,300 Mb diploid genome (about 2× size of human genome).

## Results

### The biphasic assay mechanism

TSP employs a biphasic PCR mechanism. Assays are performed using two sets of primers: a pair of primers to enrich the target sequence (locus-specific primers) and a second nested primer pair to amplify only the enriched target DNA containing the interrogated allele(s) (nested locus-specific primers). A large difference in annealing temperature between the two sets of primers separates their participation into the different stages of the reaction, thereby allowing both sets of primers to be present in the same reaction vessel. Depending on the platform used to detect the genotyping products, the benefits of this four-primer, biphasic PCR mechanism range from increased sensitivity and specificity for allele-specific primer extension chemistry to size-controlled PCR products for high resolution melt detection. These benefits result from initial enrichment of the target locus, prior to interrogation of the SNP by the nested locus-specific primers.

The use of a temperature switch during PCR enables a sequence of two amplifications to take place in a single-step, closed-tube reaction: amplification of the target sequence at a high annealing temperature, followed by detection of the harbored SNP at a low annealing temperature. This is achieved by manipulating the design of the locus-specific (LS) and nested locus-specific (NLS) primers. The LS primers are designed with a high melting temperature and to produce a PCR product greater than 400 bp that flanks the SNP allele. The NLS primers are designed with two distinct regions: a core region and a tail region and to amplify a product of less than 300 bp. The core region is complementary to sequence flanking the SNP and has a low melting temperature. The 5'-tail region is non-complementary to the DNA template and increases the melting temperature of the primer once the tail is incorporated into the PCR product. During the first phase of PCR amplification, the use of a high annealing temperature ensures that only the LS primers participate in the reaction, thereby enriching the target sequence harboring the SNP. In the second PCR phase, the annealing temperature is decreased to allow the core region of the NLS primers to hybridize to the enriched target sequence. After several PCR cycles of low-temperature annealing to allow the NLS primers to become incorporated into the DNA template, the annealing temperature is again increased. The NLS primers are present in the reaction at a sufficiently high concentration so that, in combination with their now increased melting temperature, they can compete effectively with the LS primers to result in the accumulation of the NLS primer product(s). These TSP genotyping products can be detected by a variety of single-marker methods.

### TSP genotyping by size polymorphism

**Figure 1:**
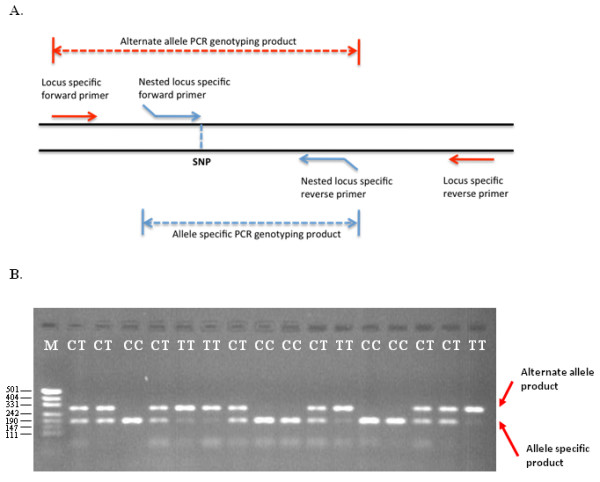
**TSP genotyping by allele-specific PCR of a CT SNP**. **A**. Schematic illustrating the position of TSP primers for allele-specific interrogation of a target region harboring a SNP and the sizes of the expected genotyping products for the two alleles. **B**. Allele-specific amplification products generated with 0.1 μM LS primers (forward 5'-GGGACATGCATGGTGGCATA and reverse 5'-GAATCTACCACCGCTCCAGCA) and 0.5 μM NLS primers designed to the C allele (forward 5'-GCGGATTGTTGCCCTAC and reverse 5'-CGTGACATTCATTTGTAGTCC). Underlined sequence indicates the non-complementary 5'-tail region. The LS forward primer was positioned 93 bp from the NLS forward primer. The presence of the C allele resulted in the accumulation of a 189 bp allele-specific PCR product (Lanes 3, 8, 9, 12 and 13), and the presence of the T (alternate) allele resulted in the formation of a 282 bp alternate allele PCR product (Lanes 5, 6, 11 and 16). Heterozygous samples resulted in the amplification of both bands, indicating the presence of the two alleles. M represents a pUC19/*HpaII *DNA size ladder.

The detection of TSP genotyping products by size polymorphism is achieved using allele-specific primer extension chemistry. In the TSP assay, allele-specificity is conferred by the terminal 3' nucleotide of the NLS forward primer and the SNP genotype is determined from the size of the TSP genotyping products. The target allele is detected by the presence of the smallest PCR product, which is amplified by the NLS forward and reverse primer pair (the allele-specific PCR product). The absence of the target allele is observed by the presence of the largest PCR fragment, which is the product of the LS forward primer and NLS reverse primer (the alternate allele PCR product). The alternate allele PCR product also acts as a positive control against a failed PCR assay. In heterozygote samples, both PCR products are amplified, thereby providing co-dominant allele detection (Figure 1).

### TSP genotyping by high resolution melt analysis

**Figure 2:**
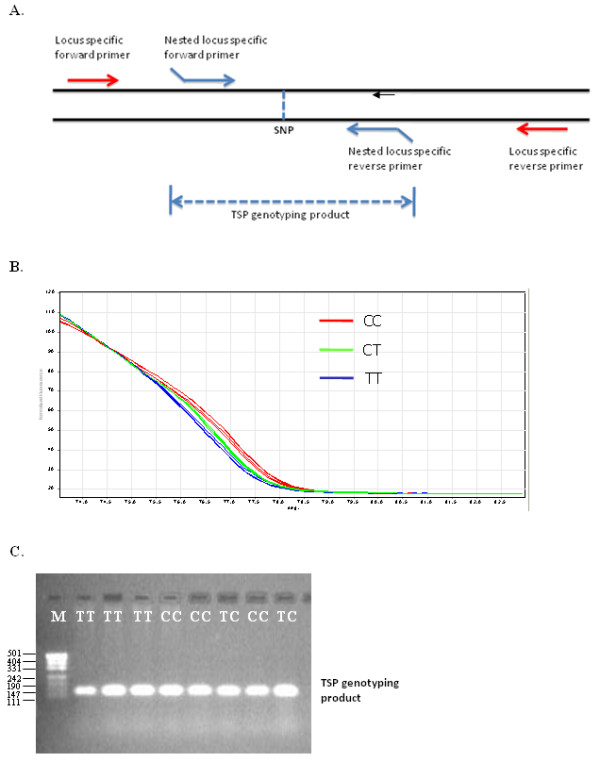
**TSP genotyping by high resolution melt (HRM) analysis of a T/C SNP**. **A**. Schematic showing the position of TSP primers for amplification of a size-defined target region harboring a SNP. **B**. HRM amplification products were generated with 0.1 μM LS primers (forward 5'-GAAGGGCGGAGCTTTGTGGA and reverse 5'-GGTGGAGCCCTGATGGCAGT) and 0.5 μM NLS primers designed to position the T/C SNP in the centre of the 142 bp amplicon (forward 5'-GGCCTCATCCTTCTGGTA and reverse 5'-GGCGCTTTTACATTTGTTAT). Underlined sequence indicates the non-complementary 5'-tail region. Following amplification, the reaction was completed with a melt gradient from 65°C to 95°C to discriminate the alleles. The presence of the T allele is represented by the blue line, the presence of the C is represented by the red line and heterozygous samples are represented by the green line. **C**. Following amplification and analysis, a subset of the reaction products were separated on a 2% agarose gel to demonstrate the specific amplification and accumulation of only the 142 bp product of the NLS primer pair. M represents a pUC19/*HpaII *DNA size ladder.

The allelic discrimination of TSP genotyping products by high resolution melt (HRM) analysis is achieved by detecting the difference in melting temperature between the PCR fragments amplified for the different alleles. The TSP assay is performed using a pair of LS primers to enrich the target sequence from the genomic template, and a pair of NLS primers to capture the harbored SNP. The NLS primers are designed without a 3' nucleotide complementary to one of the SNP alleles, and hence the assay produces a single PCR fragment of constant size regardless of the captured SNP allele(s), which is the result of the NLS primer pair. The NLS primers are designed to position the SNP towards the centre of the TSP genotyping product and to produce a PCR fragment with an optimal length for HRM analysis, which is typically between 100 and 300 bp. The advantage of this assay configuration is that the size of the TSP genotyping product and the position of the SNP within the PCR fragments can be readily adjusted to maximize allele discrimination sensitivity (Figure 2).

### The strategy for developing the biphasic PCR mechanism

During the development of TSP, the most critical steps to achieve were: (1) complete separation of the amplification step for enrichment of the target sequence from the step for interrogating the harbored SNP, and (2) efficient transition in the second phase of the reaction from continued enrichment of the target locus to the amplification of the SNP genotyping products. In developing the biphasic PCR mechanism for TSP, experiments were focused on allele-specific PCR assays designed for endpoint SNP genotyping by the detection of size polymorphism on agarose gel. Robust allele-specific PCR assays are challenging to develop due to the difficulty associated with achieving absolute allele-specificity [4,5,14,15]. Hence, allele-specific PCR assays provided a useful model for developing the biphasic PCR mechanism, since it was expected that these assays would provide the greatest challenge to achieving an efficient transition in the second phase of the reaction from enrichment of the target locus to amplification of the expected SNP genotyping products. In addition, allele-specific PCR allowed the accumulation of PCR product to be visualized on agarose gel at each stage of the reaction, which assisted the development of the optimal parameters for TSP primer design and cycling conditions.

### Defining parameters for locus-specific primer design

The first parameters investigated for the development of TSP were the melting temperature and cycle number for the LS primers. A melting temperature range of 60-65°C was chosen for designing the LS primers. This enabled the first phase of the PCR amplification to be performed at a high annealing temperature, allowing hybridization of only the LS primers, while the second phase of the PCR could be performed at a lower annealing temperature for stringent annealing of the NLS primers. Experiments were performed using 12 primer pairs that generated PCR products ranging from 500 to 1300 bp in length and a subset of 16 DNA samples. The primer concentration was maintained at 0.1 μM to ensure the locus specific primers became limiting in the second phase of amplification and to minimize primer-dimer formation.

Performing a standard thermal cycling reaction at 58°C annealing with 35 cycles of amplification produced detectable levels of PCR product of the expected size for all of the LS primer sets tested, confirming the specificity of the amplifications. To determine the number of PCR cycles required for enrichment of a target locus, the reactions were terminated at 5-cycle intervals and the products were separated on an agarose gel. Fifteen cycles was selected for enriching the target locus in the first phase of amplification, as this was the cycle threshold before which detectable levels of locus-specific product were produced when between 20 and 50 ng of genomic DNA was used as starting material. These assay parameters ensured that the locus-specific product was never amplified to a detectable level, thereby simplifying the assay output for endpoint SNP genotyping by minimising the total number of bands that would be detected in the final TSP genotyping assay.

### Defining parameters for nested locus-specific primer design

Several parameters were essential to consider when developing the NLS primers to ensure that they did not engage in the first phase of amplification, were able to compete with the LS primers once they had engaged, and did not amplify non-specifically or result in mega-priming [16].

To ensure that the NLS primers would not engage in the first phase of PCR amplification, the optimal core melting temperature of the NLS primers was investigated using 12 SNPs on a subset of 16 DNA samples. This was done empirically by designing primer cores with melting temperatures ranging from 35 to 55°C, increasing in 5°C increments. Standard thermal cycling reactions were performed with an annealing temperature of 58°C with 35 cycles of amplification using only the NLS forward and reverse primer pair to determine which primer sets did not produce an NLS product directly from genomic template. Separation of the amplification products on agarose gel revealed that NLS primers with a melting temperature of 50°C or less produced no visible product, while those with a melting temperature of 55°C produced an amplification product, irrespective of the genotype of the genomic template, implying non-specific amplification.

**Figure 3:**
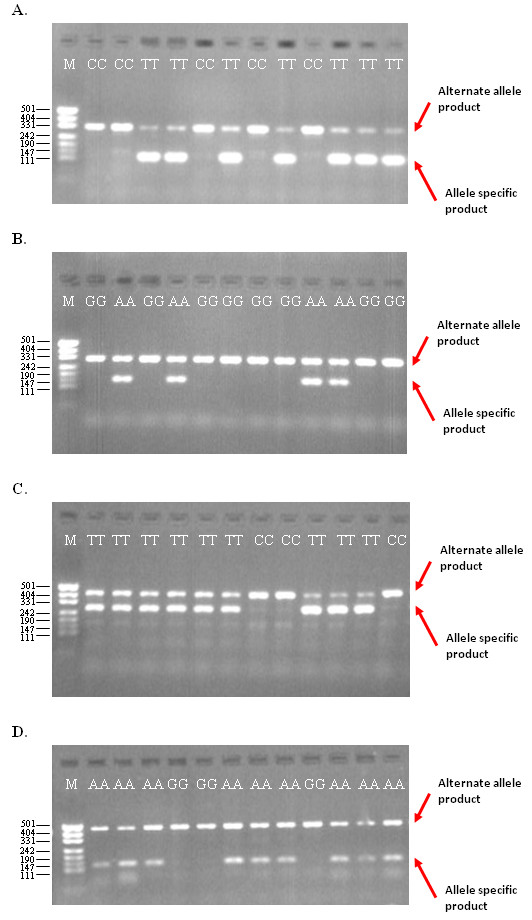
**The effect of nested locus-specific primer melting temperature on TSP genotyping efficiency**. Genomic DNA of known zygosity (homozygous wildtype or homozygous mutant) was used to test the genotyping efficiency of nested locus-specific primers with varying core melting temperatures ranging from 35°C to 50°C in TSP assays configured for allele-specific PCR. In these representative examples that target four SNP loci, an NLS primer pair with a core melting temperature of 50°C (LS forward 5'-GCCCATTCGTTTGATCAGGG and reverse 5'-CCTTTTCTTGGCGGTGATGC with NLS forward 5'-GCGCAAAATTTTAGTGTAACT and reverse 5'-CGTGATACCTGCAATGAAGT), 45°C (LS forward 5'-AGCTCCCATCGAGCTTGTGC and reverse 5'-GTTCAGCGACAGCCAACGAA with NLS forward 5'-TCGTCGAGAAGTTCCA and reverse 5'-AAAATTTGCAGGAAGTG), 40°C (LS forward 5'-GGGAGGAACAGTGCCTGCAA and reverse 5'-CCAGTCCTGGCACAACCACA with NLS forward 5'-TAGTACTGTTGCTATTGAT and reverse 5'-CTCCCACAGATGTATG) and 35°C (LS forward 5'-AGGCACTGCTGTCATGCTGG and reverse 5'-TTTTCAATCGGGCGTCTTCC with NLS forward 5'-GCATCTACAGTACCTTA and reverse 5'-TCTTCCACGGTATT) is shown (Figure 3A-D, respectively). The presence of the allele-specific genotyping product corresponds to the presence of the SNP allele. However, the presence of the alternate allele PCR product in each of these DNA samples also indicates the presence of the alternative allele, erroneously suggesting a heterozygous state. These results suggest the melting temperature of the NLS forward primer is too low to out-compete the participation of the LS forward primer. M represents a pUC19/*HpaII *DNA size ladder.

Pairs of NLS primer cores with melting temperatures of 50°C or less were tested under TSP cycling conditions (see *Materials and Methods*) in the presence of the LS forward and reverse primers using DNA samples with known genotypes. In these reactions, it was observed on agarose gel that the NLS primers were unable to effectively compete with the LS primers (Figure 3). For example, when an NLS primer pair with a core melting temperature of 45°C was tested in the TSP configuration for allele-specific PCR, the presence-absence of the 147 bp allele-specific PCR product corresponded to the correct genotype. However, each reaction also produced the 271 bp alternate allele PCR product and so incorrectly indicated a heterozygous state (Figure 3B). This suggested the NLS primers could not sufficiently outcompete amplification with the LS primer pair in the presence of the SNP allele to allow an efficient transition from enrichment to interrogation of the target allele.

**Figure 4:**
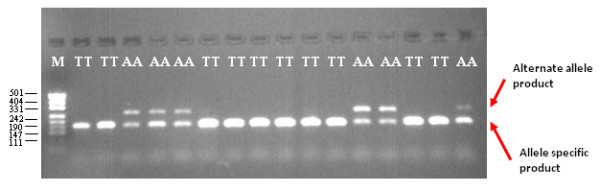
**The effect of the non-complementary 5'-tail melting temperature on TSP genotyping efficiency**. Genomic DNA of known zygosity (homozygous wildtype or homozygous mutant) was used to test the genotyping efficiency of NLS primers with a core melting temperature below 50°C and a non-complementary tail that increased the overall melting temperature from 5°C to 20°C above the core melting temperature once incorporated into PCR product. In this example, an NLS primer pair with a core melting temperature of 45°C and a non-complementary tail that increased the melting temperature to 60°C once incorporated in PCR product is shown for a TSP assay configured for allele-specific PCR (LS forward 5'-GCGTCGCAAAGACAAGCTGA and reverse 5'-CCGCAGGCGAACCTTTACAT with NLS forward 5'-CCGGGATATGTTTGGGTATCATT and reverse 5'-CCCGAACTCATGGACGCAGT). Underlined sequence indicates the non-complementary 5'-tail region. The presence of the 273 bp alternate allele genotyping product in lanes for the AA homozygotes (lanes 3, 4, 5, 12, 13 and 16) indicate the presence of the A allele. However, the presence of the 153 bp allele-specific product in these DNA samples erroneously indicates that the T allele is also present. These results suggest the 5'-tail melting temperature is too high, thereby permitting the NLS primer to engage in the first phase of amplification. M represents a pUC19/*HpaII *DNA size ladder.

To improve the ability of the NLS primers to compete with the LS primers, the addition of 5' non-complementary tails to NLS primers with a core melting temperature between 35 and 50°C was investigated. These tails increased the overall melting temperature of the NLS primers from 5 to 20°C above their core melting temperature. A tail that increased the overall melting temperature to 55°C or above resulted in the NLS primers efficiently engaging in the first phase of amplification, which resulted in non-specific allele amplification regardless of the genotype of the genomic template. Similarly, a tail that did not increase the overall melting temperature above 50°C did not allow the NLS primers to compete with the LS primers once the annealing temperature was increased in the second phase of amplification (Figure 4). A tail that increased the overall melting temperature to 53°C when added to NLS primers with a core melting temperature of 45°C was observed to not bind in the first phase of amplification and was able to out-compete the LS primers in the homozygous wildtype state. However, these primer parameters were still not sufficient to provide equal amplification for heterozygous alleles.

To improve the efficiency of the NLS primer amplification in the heterozygous state, the primer concentration was initially increased ten-fold over the LS primer pair. This greatly improved the amplification efficiency of the NLS primers. However, in allele-specific TSP assays it also resulted in the accumulation of only allele-specific PCR product, even in heterozygous samples. Decreasing the primer ratio to 5:1 balanced the accumulation of the allele-specific and alternate allele PCR products in allele-specific PCR assays during the second phase of amplification.

Overall, the final parameters selected for TSP assay design were found to be a pair of LS primers with a melting temperature of 63°C (with a range of 60-65°C) and a pair of NLS primers with a core melting temperature of 45°C (range 43-47°C) with a short 5' non-complementary tail that increased the overall melting temperature to 53°C (range 52-55°C) once incorporated into PCR product. The optimal ratio of NLS to LS primer was 5:1.

### Validation of a distinct two-stage amplification mechanism

To validate that TSP amplification was partitioned into two distinct stages, the assay was performed with a variety of primer combinations configured for allele-specific PCR or HRM detection. The accumulation of TSP genotyping products was monitored in real-time using SYBR Green and confirmed by endpoint detection on agarose gel.

**Figure 5:**
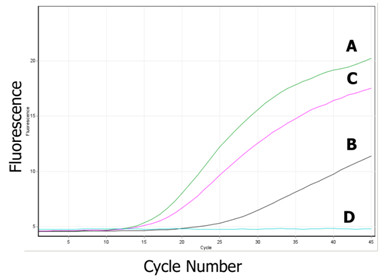
**Real-time PCR showing biphasic amplification in TSP assays**. Reactions were performed under TSP cycling conditions using **A**. 0.1 μM of LS primer pair only (forward 5'-CTACTGGAAGGCCGGCAAGC and reverse 5'-CGCATAAACCTCAACATCTGAGCA), **B**. 0.5 μM of NLS primer pair only (forward 5'-GCGTTAAGCATACAGTTTTAGTA and reverse 5'-GGGCCTGAAACCAACC). Underlined sequence indicates the non-complementary 5'-tail region. **C**. 0.1 μM of LS primer pair and 0.5 μM of NLS primer pair, and **D**. negative control reaction with 0.1 μM of LS primer pair and 0.5 μM of NLS primer pair without genomic DNA template.

In real-time PCR, assays performed under standard TSP cycling conditions with only the LS forward and reverse primers showed the rapid accumulation of product by 35 cycles (Figure 5a). This was confirmed by agarose gel electrophoresis to correspond to the amplification of the full-length target region. Without NLS primers in the reaction, there was no switch to amplification of the final TSP genotyping products (Figure 5c). In contrast, assays performed with only the NLS forward and reverse primers resulted in the delayed accumulation of PCR product until after the annealing temperature was lowered following the first 15 cycles (Figure 5b). In the final phase of TSP, an additional 10 cycles of amplification was needed to produce sufficient PCR product during the real-time analysis to detect the binding of the NLS primers to the genomic template. Endpoint detection of these products on agarose gel revealed the non-specific amplification of the allele-specific TSP genotyping product in the absence of the target allele in assays configured for allele-specific PCR. This resulted from the indiscriminate hybridization of the NLS primers directly to the genomic template in the second phase of amplification due to the absence of the LS primers to first enrich the target region. These results demonstrate an efficient partitioning of the participation of the LS and NLS primers into the first and second stages of PCR, respectively. TSP cycling performed using both LS and NLS primers showed an amplification profile similar to reactions containing only LS primer, but consistently produced less fluorescence at each cycle (Figure 5c). This reduced fluorescence corresponds to the transition from the amplification of LS product to the NLS product, and is observed because the NLS product is significantly shorter than the LS product. SYBR Green dye binds only to double-stranded DNA, producing an increase in fluorescence that is influenced by the length(s) of the PCR product(s). Endpoint agarose gel detection showed the expected TSP genotyping products in assays configured for both allele-specific PCR (Figure 1b) and HRM detection (Figure 2b). The real-time analysis therefore also demonstrates an efficient transition from the amplification of LS product to the accumulation of NLS product in the second phase of the reaction.

**Figure 6:**
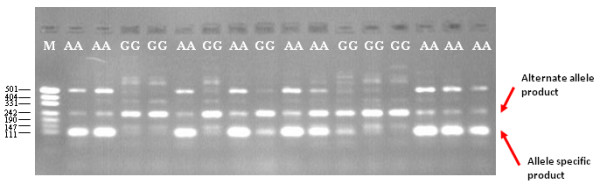
**The effect of constant annealing temperature thermal cycling on TSP genotyping efficiency**. Reactions were performed with an annealing temperature of 58°C for 35 cycles using 0.1 μM of LS primers (forward 5'-CGAGGATTGGCTCAAGACGC and reverse 5'-GCAGCGTTCTTAGGACTGGCA) and 0.5 μM of NLS primer (forward 5'-GGCCAGAGTAAGTTGCTGAA and reverse 5'-CGGTTGATCCCGTAGGTG). Underlined sequence indicates the non-complementary 5'-tail region. M represents a pUC19/*HpaII *DNA size ladder.

To further confirm the importance of the biphasic PCR mechanism for the amplification of the expected TSP genotyping products, the same set of reactions was repeated, however this time with constant cycling conditions (i.e. 35 cycles at 58°C annealing), which replicate the conditions of a published allele-specific PCR genotyping method [15]. These conditions did not produce the expected genotyping products that were seen with TSP thermal cycling (Figure 6), implying the importance of the distinct temperature switch in the TSP assay for controlling the sequential amplification of the genotyping products.

### Evaluation of TSP for endpoint SNP genotyping

To evaluate the use of TSP for endpoint SNP genotyping, assays were developed for the detection of SNP genotyping products by size polymorphism, and for detection by HRM analysis. The genotyping specificity of the assays was initially tested on a subset of DNA samples with known zygosity.

All TSP assays developed for HRM detection amplified only the size-defined region harboring the SNP deliminated by the NLS primers and produced the expected genotypes for the DNA samples tested (Figure 2), but seven of 87 TSP assays developed for allele-specific PCR resulted in the "leaky" amplification of alleles. "Leaky" amplification was defined by the presence of the NLS primer product (allele-specific PCR product) in DNA samples known to be homozygous for the alternate allele. To overcome the problem of non-specific and leaky amplification, the addition of a secondary mismatch in the NLS forward primer was investigated. The addition of a deliberate secondary mismatch near the 3' terminus of an NLS primer has been demonstrated to improve allele-specificity by destabilising the binding of the primer and improving primer extension stringency [5]. The nature and position of the secondary mismatch was calculated using WebSNAPER and TM MISMATCH functions [17,18]. Assays performed with a secondary mismatch at the n-2 or n-3 position were unable to outcompete amplification of the LS product, resulting in the amplification of both allele-specific and alternate allele PCR products for homozygous samples. Rather, redesigning the TSP markers to interrogate the alternate SNP allele proved to be the most effective method for stabilizing allele-specificity. Whilst some of the redesigned assays still produced a small amount of non-specific allele amplification, the signal-to-noise ratio was always sufficiently high to ensure unambiguous genotype assignment.

The genotyping accuracy for the 87 allele-specific PCR markers was determined in a blinded study using subsets of 376 different DNA samples, which generated a total of 11,232 data points. The range of DNA samples tested included doubled haploid, F1 and F3 plants. Doubled haploid plants were expected to be homozygous at all SNP loci, while the F1 and F3 plants were expected to contain both homozygous and heterozygous SNP loci. About 25% of the 11,232 TSP genotypes inferred heterozygosity at the SNP locus. Comparing the SNP datasets generated using the TSP markers with those produced using an independent genotyping method revealed 100% concordance in the genotype assignments. The independent genotyping data was generated using allele-specific primer extension assays performed on the BioPlex™ microsphere suspension array platform (BioRad), as described by [19] using the same DNA samples, and for the same SNP loci.

The robustness of the TSP assay was further demonstrated by amplifying DNA samples extracted using a variety of common methods (see *Materials and Methods*). Although the quality of the DNA templates was variable and often crude, the TSP assays produced the correct genotypes from the range of DNA samples, demonstrating that DNA quality was not a limiting factor. The concentration of the DNA template was the greatest variable to the success of marker amplification and accurate genotyping. The TSP assay was optimal with a starting DNA input of 100 ng or less.

## Discussion

TSP was developed to support the deployment of single-marker SNP genotyping methods in the laboratory and to reduce bottlenecks in genotyping throughput that can be caused by the requirement to optimize individual assays. TSP employs a biphasic PCR mechanism that simplifies the design of single-marker SNP genotyping assays, reduces requirements for individual assay optimisation, and increases assay specificity and genotyping accuracy. It should be possible to directly incorporate TSP into the design of assays for most current single-marker SNP genotyping methods. TSP assays are robust and support the amplification of SNP genotyping products for both the presence and absence of an allele within the range of 60 to 500 bp. Flexibility for genotyping product size provides maximal compatibility for the separation and detection of SNPs on a variety of size separation matrixes such as agarose gel, and a range of dedicated instruments such as those used for capillary electrophoresis and melt curve analysis. TSP provides further opportunities to increase genotyping throughput by assay automation, and by supporting automated data acquisition and genotype calling.

The biphasic PCR mechanism of TSP creates a method for multiplex PCR that is performed under standardized conditions, and therefore does not require optimisation of individual assays. In SNP genotyping, TSP enables the sequential amplification of a target sequence harboring a polymorphism, followed by interrogation of the SNP in a single-step assay. The biphasic mechanism allows nested PCR to occur as two distinctly separate reactions within the same vessel, in which all of the primers required for amplification are present. This is achieved through a difference in melting temperature between the two sets of primers used for the nested PCR such that the participation of each primer set can be specifically controlled by annealing temperature. The separation of the two phases of amplification was shown using QPCR (Figure 5) by the differences in cycle thresholds among primer sets and the reduction in fluorescent intensity when both sets of primers are present in the reaction, reflecting the amplification of the shorter genotyping product.

In TSP assays, separating the participation of the different primer sets by their annealing temperature provides several advantages for SNP genotyping. With fixed primer design parameters, SNP genotyping assays can be developed rapidly, and it is possible to increase the flexibility and throughout of genotyping by assaying different SNPs in the same reaction plate. The fixed primer design parameters also minimize the need to optimise individual SNP genotyping assays, since TSP has been optimized to ensure that robust biphasic PCR occurs every time. By partitioning the step for amplification of the target sequence from the step for interrogation of the SNP, primers for each stage of the assay can be optimally designed. This can improve assay specificity because primer design parameters for the first stage of the SNP genotyping process are not compromised in specificity for optimal parameters that may be different for the next stage of amplification. In other single-marker SNP genotyping methods, where multiple primers sets designed with similar melting temperature are present in the reaction, undesirable primer-primer interactions and non-specific amplification from the genomic template can result unless careful consideration is given to primer design and assay optimisation.

The application of TSP for single-marker SNP genotyping using allele-specific PCR and HRM analysis was used in the present study to illustrate the advantages of TSP over published methods based on these assay chemistries. Allele-specific PCR and HRM assays are deployed in many laboratories for endpoint SNP genotyping due to their relatively low assay cost and flexibility for meeting the changing demands of genotyping throughput that is associated with many genetic research and diagnostic applications.

Several allele-specific PCR methods for SNP genotyping are reported including ARMS, PASA, and modifications of these methods such as tetra-ARMS and bi-directional PASA (bi-PASA) [3-5]. These methods are based on the annealing and extension of a primer that is specific to the allele of interest. They commonly employ two flanking primers to amplify the target sequence harboring the polymorphism and a third primer adjacent to the polymorphism to interrogate the SNP. The addition of a fourth primer to the assay allows the interrogation of both SNP alleles and therefore the presence of heterozygosity to be detected in a single assay. The main limitation to these methods, especially for assays based on the four-primer system, is a complicated primer design process (due to the requirement to design primers that do not result in undesirable primer-primer, or primer-template interactions that comprise assay specificity), the need to optimise individual assays to achieve allele-specific amplification (usually by individual adjustment of the concentration of each primer in the assay), and variable amplification efficiency due to differences in product length (caused by PCR competition effects). Significant effort has been invested into ways to improve the specificity of allele-specific PCR through modification of the allele-specific primers including the introduction of a secondary mismatch near the 3'-terminus of the primer, and the use of modified nucleotides such as LNA and phosphothioate linkages [20,21], but these add to the complexity of assay design and cost.

The configuration of TSP for allele-specific PCR simplifies assay primer design and reduces the requirement for optimisation of individual assays. The biphasic PCR mechanism avoids undesirable primer-primer interactions between the different sets of primers by separating their participation into different stages of the reaction. It also prevents undesirable participation of the NLS primers in the early stages of the reaction, which can compromise assay specificity and genotyping accuracy. This latter feature reduces the possibility of mis-priming, which can occur when amplifying and enriching a SNP directly from genomic template. Mis-priming compromises genotyping accuracy because PCR product for the alternate SNP allele can accumulate even in the absence of that allele, a phenomenon known as allele leakage. The ease for designing allele-specific PCR assays using TSP was demonstrated by developing 87 TSP markers, whose assays are performed under identical reaction conditions. Only seven of these markers showed SNP allele leakage that compromised genotyping accuracy, and in each case this was resolved by targeting the alternate SNP allele. High genotyping accuracy was demonstrated by 100% concordance of the 11,232 TSP genotypes generated in a blinded study with an independent genotyping method. Moreover, genotyping accuracy was maintained across samples with a range of DNA quality, indicating the tolerance of TSP to samples prepared using different DNA extraction methods. The only factor found to affect genotyping accuracy was overloading the assay with DNA (> 100 ng), a result consistent with what has been found for other allele-specific PCR genotyping methods, for which a low DNA starting concentration is best [14,15].

TSP confers advantages over current allele-specific PCR methods for endpoint SNP genotyping. In addition to the benefits of speed of assay design and genotyping throughput, TSP provides the flexibility to achieve codominant genotyping whilst targeting only one allele for primer design, since the amplification of an allele-specific, alternate allele, or both genotyping products indicates the presence of homozygous and heterozygous alleles (Figure 1). It also provides a mechanism to distinguish between the absence of the target allele and a failed reaction, since genotyping products are always produced. Perhaps the most important advantage of TSP for allele-specific PCR is flexibility for assay design. In a separate study, we showed the same assay specificity and genotyping accuracy could be achieved using a three-primer system, in which only one nested locus-specific primer is present [22]. In these assays, the nested locus-specific reverse primer was removed. The advantage of a three- versus four-primer TSP design for allele-specific PCR depends on the sequence context flanking the SNP and the requirement for a specific PCR product size for the alternate allele PCR genotyping product, which depends on the platform used for detecting the SNP genotyping products. These TSP assay configurations for allele-specific PCR support rapid, low-cost endpoint SNP genotyping, since they are not reliant on fluorescently-labeled primers or probes and they do not require individual assay optimisation to accurately assign a genotype. Rather, the standardized assay conditions and simple codominant genotyping data output provides opportunities to automate assay setup, data acquisition and genotype calling.

Similarly, the configuration of TSP for endpoint SNP genotyping using HRM illustrates the benefits of incorporating TSP into the design of SNP genotyping methods that are based on detecting changes to the physical properties of DNA. Detection of the polymorphism by HRM relies on the differential melting of amplicons based on a single base difference. The detection sensitivity is affected by both the size of the PCR fragment and the position of the SNP within the amplicon [23]. However, it can be difficult to capture the polymorphism at an optimal position within the PCR fragment to achieve maximal detection sensitivity. This can result from unfavorable flanking sequence composition and high sequence similarity between related genes; a problem often encountered for the assay of SNPs in multi-gene families and duplicated genes. In such instances, TSP can provide an advantage for endpoint SNP genotyping, since the ability of the biphasic mechanism to perform nested PCR in a single-step assay eliminates the requirement for a separate assay to preamplify the target sequence harboring the SNP. By enriching the target sequence from the genomic template in the first phase of the TSP reaction, a set of nested locus-specific primers designed for maximal detection sensitivity can be used without concern for interference by other factors that would otherwise confound SNP detection. The design of these TSP assays, however relies on the ability to design LS primers for specifc amplification of the gene of interest. In the present study, all of the TSP genotyping assays designed for endpoint HRM detection of the SNP produced robust amplification of the target sequence as a single genotyping product (Figure 2), demonstrating the fidelity required for accurate SNP genotyping. TSP could also be useful in SNP genotyping methods that are based on enzymatic modification, as these also require that the SNP be positioned appropriately within the PCR fragment (in order to produce scorable restriction fragments).

TSP was developed and optimised using genomic DNA from cultivated barley (*Hordeum vulgare *L.), an agriculturally important crop with a 5,300 Mb diploid genome about twice the size of the human genome. Assays were performed using between 20 and 100 ng of genomic DNA without loss of sensitivity or specificity. Adapting TSP for use in another species, or with a different amount of starting genomic template only requires optmisation of the PCR cycle number in the first phase of the assay. Once the optimal cycle number is determined, all other TSP primer design parameters and reaction conditions remain the same. TSP has been successfully deployed for SNP genotyping in zebrafish [24], human [25] and mouse (unpublished data) using allele-specific PCR. These assays were performed using the TSP cycling conditions described for barley, and between 20 and 50 ng of genomic DNA as starting template.

Most SNP genotyping methods have been developed to assay diploid organisms. In polyploid organisms, including many plants, genotyping is complicated by the presence of two or more gene copies in the nucleus. Selective PCR amplification using primers specific to one or another copy of the duplicated locus [26] is frequently used as a strategy to overcome this complication. However, this approach cannot be easily scaled-up and used for developing high-throughput genotyping assays. A potential advantage of TSP is its ability to address the challenges of SNP genotyping in polyploid genomes by enabling both the amplification and interrogation of a specific gene copy harboring a SNP. The successful application of a single-step assay in a polyploid system requires complete separation of the participation of the primer set for genome-specific (or gene copy-specific) amplification from the participation of the primers for interrogating the SNP. The gene copy harboring the SNP must be enriched substantially over other copies of the gene before the allele-specific (nested) primers are able to participate. It is only then that amplification from these allele-specific primers can be diagnostic for the presence-absence of the target SNP. The utility of TSP for SNP genotyping in polyploid plant species using allele-specific PCR and HRM detection methods is now being investigated in our laboratory.

## Conclusion

TSP permits the rapid construction of robust and convenient endpoint SNP genotyping assays. These assays are carried out in a single-step format under standardized conditions, and require minimal optimization of individual assays. The utility of TSP for accurate and codominant SNP genotyping using allele-specific PCR and high resolution melt analysis was demonstrated. Theoretically, TSP can be directly incorporated into the design of assays for most current single-marker SNP genotyping methods to provide enabling advances that simplify assay design and development, increase assay throughput, and improve assay automation and genotyping calling. TSP has broad applications and can be deployed in animal and plant species.

## Methods

### Plant materials and DNA extraction

The development of TSP was undertaken using genomic DNA extracted from cultivated barley (*Hordeum vulgare *L.), an agriculturally important crop with a large (5300 Mb), diploid genome. The barley lines used to assess TSP marker amplification and for SNP genotyping were based on a collection of released varieties, breeding materials and mapping populations obtained from the University of Adelaide barley breeding program and included doubled haploid, F_1 _and F_3 _progeny. These lines represented all types of zygosity and therefore could be used to confirm the ability of TSP markers to detect heterozygous SNP loci. DNA samples were prepared from leaf material of individual plants using three extraction methods to assess the effect of DNA quality on TSP amplification and genotyping accuracy. High quality DNA, free of RNA and cellular protein, was extracted using the phenol-chloroform method described by Devos et al. [27]. Medium quality DNA, containing RNA and a moderate level of cellular protein, was extracted using the salt-based method described by Benito et al. [28]. Poor quality DNA, containing large amounts of soluble cellular protein and RNA, was prepared using the sodium hydroxide method described by Paris and Carter [29]. The latter method produced DNA of quality suitable for immediate use in PCR but which underwent rapid degradation over a period of days.

### Primer design

All primers were designed with Primer3 v.0.4.0 and NetPrimer software [30,31]. The LS primer design parameters included a Tm of 60-65°C (optimal 63°C) and a product size of greater than 400 bp. The NLS primer design parameters included a core region with a Tm of 43-47°C (optimal 45°C) and a non-complementary 5' tail region that increased the overall primer Tm to 52-55°C (optimal 53°C) once incorporated into PCR product. For TSP genotyping by size polymorphism, the primers were positioned so the NLS forward primer was at least 50 bp from the LS forward primer to ensure reliable separation of the allele-specific and alterate allele genotyping products on agarose gel. For TSP genotyping by HRM analysis, the NLS forward and reverse primers were designed to position the SNP towards the centre of the genotyping product and to create a PCR product size optimal for HRM analysis.

### TSP genotyping by size polymorphism

TSP assays were performed under standardized reaction conditions. PCR was performed in a 4 μl reaction mixture containing 1× PCR buffer (16 mM (NH_4_)_2_SO_4_, 0.01% Tween-20, 100 mM Tris-HCl, and pH 8.3), 1.5 mM MgCl_2_, 100 ng/μl bovine serum albumin Fraction V (Sigma Aldrich), 0.2 mM dNTP, 0.1 μM each of forward and reverse LS primer, 0.5 μM of forward and reverse NLS primer (unless otherwise stated), 0.15 U *Tfi *DNA polymerase (Invitrogen) and between 10-50 ng genomic DNA desiccated by evaporation. Amplification was performed in 384-well PCR microplates. Following an initial denaturation step at 95°C for 3 min, TSP amplification was performed for a total of 35 cycles with the profile: 15 cycles of 95°C for 30 s, 58°C for 30 s, 72°C for 60 s to enrich the target locus harboring the SNP of interest (hereafter referred to as the first reaction phase). The second reaction phase consisted of 5 cycles of 95°C for 10 s and 45°C for 30 s, followed by 15 cycles each consisting of 95°C for 10 s, 53°C for 30 s and 72°C for 5 s. The reaction products were separated by electrophoresis on a 2% agarose gel and visualized by ethidium bromide staining [32].

### Real-time PCR and TSP genotyping by HRM analysis

Real-time PCR amplification was conducted using 1× iQ SYBR Green Supermix (BioRad) with 0.1 μM forward and reverse LS primers, 0.5 μM forward and reverse NLS primers, and 20 ng of DNA in a 10 μl reaction volume. Thermocycling was performed on a Rotor-Gene 6000 (Corbett) instrument using the PCR profile described above with a single fluorescent reading taken at the end of each cycle. The reaction was completed with a melt curve analysis consisting of a melt gradient from 65°C to 95°C, increasing in 1°C increments every 5 s, to discriminate the alleles and to confirm the specificity of amplification and lack of primer-dimer in the real-time PCR reaction.

## Abbreviations

NLS: nested locus-specific; HRM: high resolution melt; LS: locus-specific; SNP: single nucleotide polymorphism; TSP: temperature-switch PCR.

## Competing interests

An international patent has been filed for TSP, and the authors TT and MH are inventors on the patent application. The technology is available for research-only purposes. Information about licenses for conducting TSP for non-research and commercial purposes is available from the Molecular Plant Breeding CRC http://www.molecularplantbreeding.com.

## Authors' contributions

TT and MJH jointly developed the TSP method and wrote the manuscript. DEM provided critical review of the manuscript. All authors read and approved the final manuscript.
